# The Effects of Internet-Based Cognitive Behavioral Therapy for Suicidal Ideation or Behaviors on Depression, Anxiety, and Hopelessness in Individuals With Suicidal Ideation: Systematic Review and Meta-Analysis of Individual Participant Data

**DOI:** 10.2196/46771

**Published:** 2023-06-26

**Authors:** Lasse B Sander, Marie Beisemann, Philipp Doebler, Hannah Moon Micklitz, Ad Kerkhof, Pim Cuijpers, Philip Batterham, Alison Calear, Helen Christensen, Eva De Jaegere, Matthias Domhardt, Annette Erlangsen, Ozlem Eylem-van Bergeijk, Ryan Hill, Charlotte Mühlmann, Marie Österle, Jeremy Pettit, Gwendolyn Portzky, Lena Steubl, Bregje van Spijker, Joseph Tighe, Aliza Werner-Seidler, Rebekka Büscher

**Affiliations:** 1 Medical Psychology and Medical Sociology Faculty of Medicine University of Freiburg Freiburg Germany; 2 Department of Statistics Technical University of Dortmund Dortmund Germany; 3 Department of Clinical, Neuro and Developmental Psychology Amsterdam Public Health Research Institute Vrije Universiteit Amsterdam Amsterdam Netherlands; 4 Centre for Mental Health Research College of Health and Medicine The Australian National University Canberra Australia; 5 Black Dog Institute University of New South Wales, Sydney New South Wales Australia; 6 School of Medicine University of New South Wales, Sydney New South Wales Australia; 7 Department of Head and Skin Flemish Centre of Expertise in Suicide Prevention Ghent University Ghent Belgium; 8 Department of Clinical Psychology and Psychotherapy Ulm University Ulm Germany; 9 Danish Research Institute for Suicide Prevention Mental Health Centre Copenhagen Copenhagen Denmark; 10 Faculty of Behavioral and Social Sciences Webster University Leiden Leiden Netherlands; 11 Department of Psychology Louisiana State University Baton Rouge, LA United States; 12 Department of Psychology Center for Children and Families Florida International University Miami, FL United States

**Keywords:** meta-analysis, internet-based cognitive behavioral therapy, suicidal ideation, anxiety, depression, hopelessness, depressive, mental health, systematic review, review method, suicide, suicidal, psychotherapy, CBT, cognitive behavioral therapy

## Abstract

**Background:**

Suicide is a global public health problem. Digital interventions are considered a low-threshold treatment option for people with suicidal ideation or behaviors. Internet-based cognitive behavioral therapy (iCBT) targeting suicidal ideation has demonstrated effectiveness in reducing suicidal ideation. However, suicidal ideation often is related to additional mental health problems, which should be addressed for optimal care. Yet, the effects of iCBT on related symptoms, such as depression, anxiety, and hopelessness, remain unclear.

**Objective:**

We aimed to analyze whether digital interventions targeting suicidal ideation had an effect on related mental health symptoms (depression, anxiety, and hopelessness).

**Methods:**

We systematically searched CENTRAL, PsycInfo, Embase, and PubMed for randomized controlled trials that investigated guided or unguided iCBT for suicidal ideation or behaviors. Participants reporting baseline suicidal ideation were eligible. Individual participant data (IPD) were collected from eligible trials. We conducted a 1-stage IPD meta-analysis on the effects on depression, anxiety, and hopelessness—analyzed as 2 indices: symptom severity and treatment response.

**Results:**

We included IPD from 8 out of 9 eligible trials comprising 1980 participants with suicidal ideation. iCBT was associated with significant reductions in depression severity (b=−0.17; 95% CI −0.25 to −0.09; *P*<.001) and higher treatment response (ie, 50% reduction of depressive symptoms; b=0.36; 95% CI 0.12-0.60; *P*=.008) after treatment. We did not find significant effects on anxiety and hopelessness.

**Conclusions:**

iCBT for people with suicidal ideation revealed significant effects on depression outcomes but only minor or no effects on anxiety and hopelessness. Therefore, individuals with comorbid symptoms of anxiety or hopelessness may require additional treatment components to optimize care. Studies that monitor symptoms with higher temporal resolution and consider a broader spectrum of factors influencing suicidal ideation are needed to understand the complex interaction of suicidality and related mental health symptoms.

## Introduction

### Background

Suicide continues to be a major public health concern globally. Each year, more than 700,000 people die from suicide, making it the second-leading cause of death among adolescents and young adults [1]. Among various risk factors, suicidal ideation is one of the strongest predictors of subsequent suicidal behavior [2]. Studies have demonstrated that severe and pervasive ideation is associated with death by suicide [3], and even passive ideation, such as a wish to die, has been identified as a risk factor for death by suicide [4].

To overcome treatment barriers and increase the scalability of treatment measures for people with suicidal ideation [5,6], digital interventions specifically tailored to this target group have been developed and evaluated in the last decade [7-10]. A recent meta-analysis of individual participant data (IPDMA) of randomized controlled trials (RCTs) demonstrated that digital interventions based on cognitive behavioral therapy (CBT) were effective in reducing suicidal ideation with an effect size of b=−0.25 (95% CI −0.32 to −0.17) [11]. Prior research suggested that it is pertinent to address suicidality head-on, as “indirect interventions” (eg, digital interventions for depression) have been shown to be less effective in reducing suicidal ideation in digital interventions [7] as well as in face-to-face treatments [12].

However, suicidal ideation is a multifaceted condition that occurs both outside of and within many mental health disorders and physical health conditions [13-19]. Therefore, it should be considered embedded and not detached from other symptoms. In complex network theories, suicidal ideation is considered to be a consequence of multiple factors, including symptoms of mental health, that interact with one another in a network structure [20,21]. Following the network theory, the effective treatment of suicidal ideation may therefore also be expected to have an impact on related symptoms. Potential effects on related symptoms are of clinical relevance for personalized treatment planning: As people with suicidal ideation often have a diagnosis of one or more mental disorders [17,22], the selection of additional treatment components to provide optimal care may vary in accordance with the differential effectiveness of an initial intervention [23]. Prior systematic reviews and meta-analyses of face-to-face CBT for suicidal ideation and self-harm indicated small effects on depression and hopelessness [24-26].

### Objective

The aim of this study was to analyze whether RCTs targeting suicidal ideation or behaviors had an effect on related mental health symptoms in people with suicidal ideation. Included trials were identified in a recent IPDMA [11]. While meta-analyses of aggregated data can provide overall effect estimates, IPDMAs come with the advantage of allowing standardized analyses across all trials (eg, the same imputation strategy) and increased power, potentially leading to a more precise effect estimate. In addition, individual participant data (IPD) can be checked for accuracy [27]. Thus, IPDMAs are considered to be the gold standard for evidence synthesis [28].

## Methods

This report adheres to the PRISMA (Preferred Reporting Items for Systematic Reviews and Meta-Analyses) statement for IPD systematic reviews. The review was preregistered with the Open Science Framework (OSF). The PRISMA checklist for IPD systematic review can be found in Multimedia Appendix 1.

### Eligibility Criteria

Textbox 1 shows an overview of the study inclusion criteria. Participants were eligible if they reported suicidal ideation at baseline. Studies had to investigate stand-alone internet-based cognitive behavioral therapy (iCBT; including third-wave therapies, eg, dialectic behavioral therapy) that directly addressed suicidal ideation or behaviors. iCBT interventions were defined as internet or mobile apps that include multiple components of CBT in several modules. Interventions could include some human support, for example, written feedback. Control conditions could be treatment as usual, no intervention, other active or passive control groups, or waitlist conditions. Studies had to report a quantitative measure of suicidal ideation. We included RCTs published in peer-reviewed journals. There were no restrictions on language or publication dates. We excluded blended care interventions (ie, interventions where iCBT is only an adjunct to face-to-face therapy) and interventions exclusively targeting stigma or help seeking, as well as interventions directed at gatekeepers (eg, teachers).

**PICOS (participants, interventions, comparators, outcomes, and study design) elements of the study inclusion criteria.**

**Participants**

- Individuals with suicidal ideation

**Interventions**

- Digital (internet- or mobile-based) cognitive behavioral therapy interventions for suicidal ideation or behaviors with or without human support (guidance)

**Comparators**

- Treatment as usual
- No intervention
- Waitlist
- Other active or passive control groups

**Outcomes**

- Secondary outcomes of primary trials assessed by diagnostic interviews and self-reported and clinician-rated scales

**Study design**

- Randomized controlled trials published in peer-reviewed journals in all languages

### Search Strategy

We systematically searched CENTRAL, PsycInfo, Embase, and PubMed from inception to January 30, 2022, using predefined search terms. The full electronic search strategy can be found in Multimedia Appendix 2. Two independent reviewers (RB and HMM) screened titles and abstracts for relevant studies. In the next step, they screened the full texts of identified trials. Conflicting decisions were discussed with a third researcher (LBS). We performed reference searches using Web of Science.

### Data Collection

Authors of primary trials were asked to provide the anonymized raw IPD. We conducted data checks, comparing published data with the IPD. Two independent reviewers (RB and HMM) transferred data from the raw IPD into a combined file using a consistent scheme for all trials; any discrepancies were resolved in discussion. Sociodemographic and clinical variables were extracted for all time points, and authors resolved any queries. Furthermore, 2 independent reviewers (Hanna Helfrich and RB) extracted data items from the published papers. The study-level data were used for the conventional meta-analysis and data integrity checks of the extracted IPD; no discrepancies were found.

### Risk of Bias

Risk of bias was evaluated for the 3 outcomes based on the revised Cochrane Risk of Bias Tool for RCTs. Risk of bias was assessed in the following domains: (1) bias arising from the randomization process, (2) bias due to deviations from intended interventions, (3) bias due to missing outcome data, (4) bias in measurement of the outcome, and (5) bias in selection of the reported result. We did not evaluate the items referring to blinding of outcome assessors (signaling questions 4.3 to 4.5) since the tool defines participants as outcome assessors for outcomes that cannot be measured without incorporating the participant’s perspective (also in clinician-rated outcomes). The participants are likely to know about their condition (4.3) and the participants’ reports might be influenced by this knowledge (4.4 and 4.5) across all studies and outcomes; thus, these items would lead to ceiling effects of the rating. We did not assess publication bias because the investigated outcomes are secondary outcomes of trials and therefore less likely to have an impact on the publication. A previous IPDMA on the same set of trials did not find indications for publication bias [11].

### Statistical Analysis

We performed a 1-stage IPDMA, investigating the effects of iCBT on all related outcomes, which were assessed in a sufficient number of primary studies. This was the case for depression, anxiety, and hopelessness. The 1-stage approach combines data from all studies in 1 hierarchical model. Unlike 2-stage IPDMA, which performs a conventional meta-analysis after calculating effect sizes for each trial separately, 1-stage IPDMA produces less biased estimates [29]. The IPDMA was conducted with 2 indices for each outcome: first, a continuous measure on severity (ie, changes from baseline), and second, the response rate (ie, 50% symptom reduction compared to baseline, according to Frank and colleagues [30]). We corrected for multiple testing within the 2 indices using the Bonferroni correction. The change scores were scaled to their study-specific variance for comparability between different scales. Missing observations were imputed study-wise using multiple imputations (m=100) relying on predictive mean matching.

For the continuous measure of symptom severity, a multilevel linear regression was performed. For the response rate, a logistic multilevel regression was conducted. To account for the hierarchical structure of the data (ie, patients nested in studies), we modeled a random intercept in each of the IPDMA models. The treatment effect was modeled either as fixed (for a homogenous effect across studies) or random (for a heterogeneous effect across studies) based on model comparisons with likelihood ratio tests analogous to the Q test.

The following sensitivity analyses were conducted. First, a sensitivity analysis was performed only using data from participants who completed all assessments. Second, a conventional random-effects meta-analysis was conducted for the continuous outcome measures (ie, depression, anxiety, and hopelessness). We calculated a between-group effect size (Hedges *g*) based on changes from baseline to postintervention in each condition. The conventional meta-analysis was used to examine potential differences between the trials that provided data for the IPDMA and those that did not, using a subgroup analysis.

The analyses were performed with R (version 3.6.1; R Foundation for Statistical Computing). This study was preregistered with the OSF. The analysis scripts will be provided in OSF with publication.

## Results

### Study Selection

**Figure 1:**
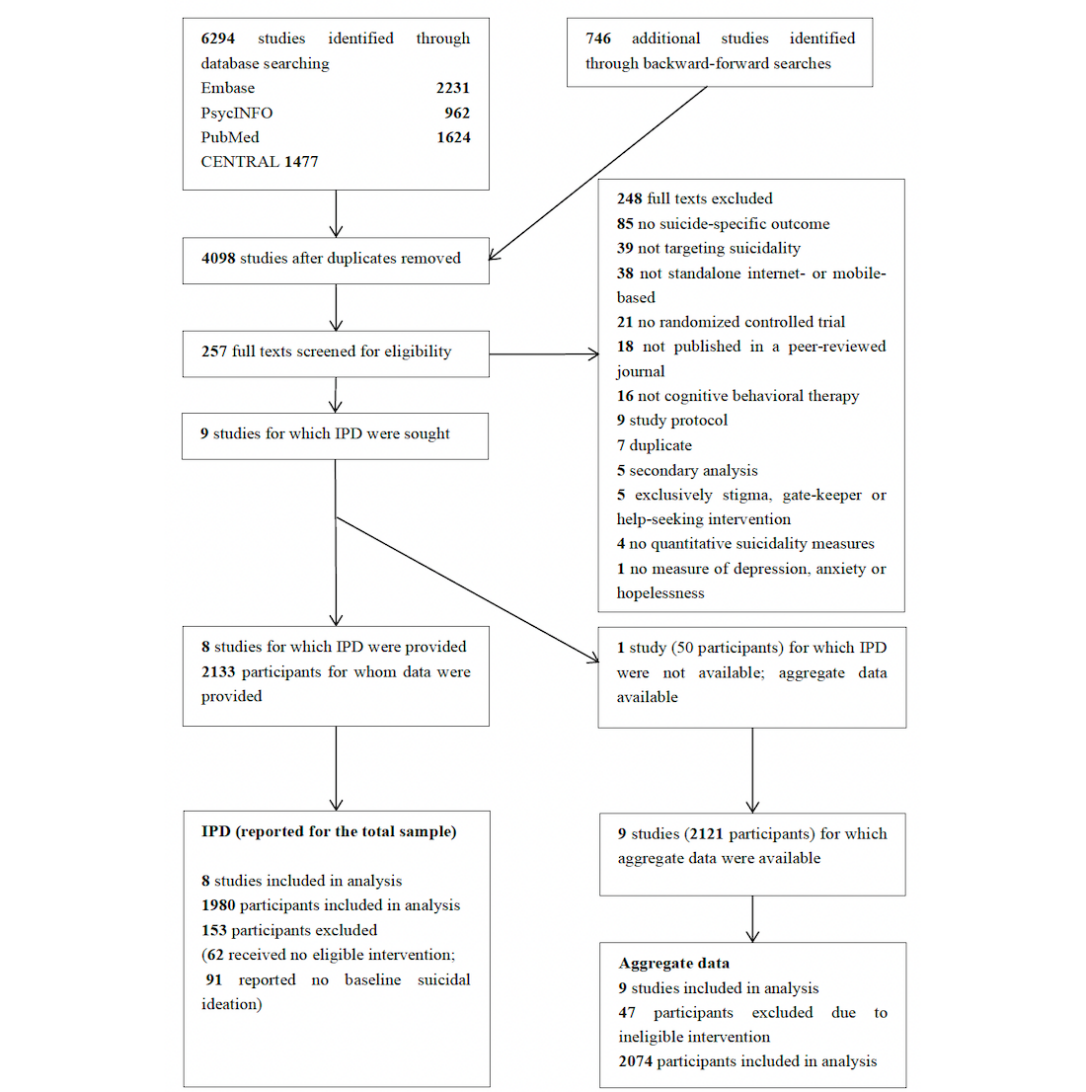
PRISMA (Preferred Reporting Items for Systematic Reviews and Meta-Analyses) flowchart. IPD: individual participant data.

The study selection process is shown in Figure 1. In total, 4098 unique records were identified in the database searches; 9 eligible studies were identified, and IPD were obtained from 8 (89%) trials [31-38], whereas in 1 case, data were not available [39]. After excluding 153 (7.2%) ineligible participants out of 2133 participants (reasons are given in Figure 1), we included 1980 participants in the IPDMA. A total of 990 participants (50%) were assigned to iCBT conditions, and 990 (50%) were assigned to control groups.

### Study, Intervention, and Participant Characteristics

For an overview of study characteristics, see Table 1. Most study populations consisted of adults, whereas 2 studies [36,39] included adolescents. In 6 studies, participants were recruited from the general population; 3 studies included specific target groups, that is, Aboriginal and Torres Strait Islander youth (Australia) [32], Turkish migrants [35], and school students [39]. One trial used a mobile-based intervention [32]; the majority of studies used internet-based programs. In total, 6 studies investigated unguided interventions [31-33,36-38]. In 3 studies, guidance was delivered by inviting participants to text the team in case of questions [34], by interacting and providing written feedback via a message board [39], or by providing regular meetings via Skype or email [35]. In total, 5 studies investigated adapted versions of the same intervention [31,34,35,37,38]. This intervention is based on CBT techniques including psychoeducation, problem-solving, cognitive restructuring, and worry scheduling [40]. One intervention provided a personalized combination of modules based on symptom measurements [33]; the other interventions were not personalized. The control condition was waitlist in 6 trials [31,32,34-37]; three studies used active control groups [33,38,39], including web-based attention control [33,38] or treatment as usual [39], which was contact with school staff and potential side treatments. The number of digital modules ranged from two [36] to ten [33]; the maximum time participants were recommended to spend using the intervention ranged from one [36] to 21 hours [31,34,37,38].

In this IPDMA, 1347 (68.5%) out of 1966 participants were female. The mean age of participants was 36.2 (SD 13.5) years. A total of 887 (48.1%) out of 1844 participants reported at least 1 previous suicide attempt, and 906 (56.3%) out of 1608 participants were in additional psychological or psychiatric treatment at baseline (Table 2).

**Table 1:** Study characteristics.

Study	Participant, n	Target group	Eligible age group (years)	Comparison	iCBT^a^ weeks, n (modules, n)	Included measure of depression	Included measure of anxiety	Included measure of hopelessness	IPD^b^
Batterham et al [33] (2018)	132^c^	General population (young adults)	18+ (initially 18-25)	Unguided iCBT (web-based) Active web-based intervention	2 (10)	PHQ-9^d^	GAD-7^e^	—^f^	Yes
De Jaegere et al [37] (2019)	724	General population (adults)	18+	Unguided iCBT (web-based) Waitlist	6 (6)	BDI^g^	HADS-A^h^	BHI^i^	Yes
Eylem et al [35] (2021)	18	Turkish migrants (adults)	18+	Guided iCBT (web-based) Waitlist	6 (6)	BDI	—	BHI	Yes
Hetrick et al [39] (2017)	50	School students (adolescents)	13-19	Guided iCBT (web-based) + TAU TAU	10 (8)	RADS^j^	MASC^k^	BHI	No
Hill and Pettit [36] (2019)	80	General population (adolescents)	13-19	Unguided iCBT (web-based) Waitlist	2 (2)	RADS	—	—	Yes
Mühlmann et al [34] (2021)	402	General population (adults)	18+	Guided iCBT (web-based) Waitlist	6 (6)	HAM^l^	—	BHI	Yes
Tighe et al [32] (2017)	61	Australian Aboriginal and Torres Strait Islander youth (young adults)	18+ (initially 18-35)	Unguided iCBT (app) Waitlist	6 (3)	PHQ-9	—	—	Yes
van Spijker et al [31] (2014)	236	General population (adults)	18+	Unguided iCBT (web-based) Waitlist	6 (6)	BDI	HADS-A	BHI	Yes
van Spijker et al [38] (2018)	418	General population (adults)	18-65	Unguided iCBT (web-based) Active web-based intervention	6 (6)	CESD^m^	GAD-7	BUHS^n^	Yes

^a^iCBT: internet-based cognitive behavioral therapy.

^b^IPD: individual participant data.

^c^The total number of participants does not include the ineligible static intervention condition (third intervention arm with an additional n=62, excluded from our analyses).

^d^PHQ-9: Patient Health Questionnaire-9.

^e^GAD-7: Generalized Anxiety Disorder.

^f^Not available.

^g^BDI: Beck Depression Inventory.

^h^HADS-A: Hospital Anxiety and Depression Scale—Anxiety.

^i^BHI: Beck Hopelessness Inventory.

^j^RADS: Reynolds Adolescent Depression Scale.

^k^MASC: Multidimensional Anxiety Scale for Children.

^l^HAM: Hamilton Depression Rating Scale.

^m^CESD: Centre for Epidemiological Studies Depression Scale.

^n^BUHS: Burns Hopelessness Scale.

**Table 2:** Baseline participant characteristics.^a^

		Study^b^, n	iCBT^c^ conditions		Control conditions		Total sample	
			Participant^d^, n	Value	Participant, n	Value	Participant, n	Value
**Depression, mean (SD)**								
	BDI^e^	3	416	32.41 (10.88)	390	32.74 (11.31)	806	32.57 (11.08)
	CESD^f^	1	206	40.78 (9.54)	210	39.81 (9.64)	416	40.29 (9.59)
	HAM^g^	1	194	13.59 (4.06)	205	12.69 (4.28)	399	13.13 (4.20)
	PHQ-9^h^	2	69	11.03 (5.46)	61	12.46 (5.21)	130	11.70 (5.37)
	RADS^i^	1	33	26.09 (4.13)	32	27.16 (4.59)	65	26.62 (4.36)
**Anxiety, mean (SD)**								
	GAD-7^j^	2	252	12.12 (5.58)	249	12.37 (5.29)	501	12.24 (5.43)
	HADS-A^k^	2	396	12.67 (4.09)	375	12.63 (4.25)	771	12.65 (4.16)
**Hopelessness, mean (SD)**								
	BHI^l^	4	605	14.76 (3.52)	591	14.63 (3.80)	1196	14.70 (3.66)
	BUHS^m^	1	206	13.13 (4.90)	210	12.58 (4.73)	416	12.85 (4.81)
**Suicidal ideation, mean (SD)**								
	BSS^n^	5	662	18.34 (7.52)	663	18.55 (7.22)	1325	18.44 (7.37)
	SIDAS^o^	2	252	24.08 (12.41)	249	22.51 (11.95)	501	23.30 (12.20)
	DSI-SS^p^	1	23	3.70 (1.69)	22	3.14 (1.42)	45	3.42 (1.57)
Age (years), mean (SD)		6	913	35.97 (13.58)	926	36.37 (13.37)	1839	36.17 (13.47)
History of suicide attempts, n (%)		6	918	440 (47.9)	926	447 (48.3)	1844	887 (48.1)
Female gender, n (%)		8	985	670 (68)	981	677 (69)	1966	1347 (68.5)
Secondary education or higher, n (%)		6	838	751 (89.6)	821	748 (91.1)	1689	1499 (90.4)
Married or living with partner, n (%)		4	706	204 (28.9)	697	192 (27.5)	1403	396 (28.2)
Employed, n (%)		5	321	210 (65.4)	360	235 (65.3)	681	445 (65.3)
Current treatment, n (%)		5	808	467 (57.8)	800	439 (54.9)	1608	906 (56.3)

^a^These descriptive analyses are based on complete observations (unimputed data).

^b^The number of studies.

^c^iCBT: internet-based cognitive behavioral therapy.

^d^The total number of participants who provided data on the respective variable.

^e^BDI: Beck Depression Inventory.

^f^CESD: Centre for Epidemiological Studies Depression.

^g^HAM: Hamilton Depression Rating Scale.

^h^PHQ-9: Patient Health Questionnaire-9.

^i^RADS: Reynolds Adolescent Depression Scale.

^j^GAD-7: Generalized Anxiety Disorder.

^k^HADS-A: Hospital Anxiety and Depression Scale—Anxiety.

^l^BHI: Beck Hopelessness Inventory.

^m^BUHS: Burns Hopelessness Scale.

^n^BSS: Beck Scale for Suicide Ideation.

^o^SIDAS: Suicidal Ideation Attributes Scale.

^p^DSI-SS: Depression Symptom Index—Suicidality Subscale.

### Effects on Depression, Anxiety, and Hopelessness

The severity of depression was reduced in iCBT conditions compared with control conditions (b=−0.17; 95% CI −0.25 to −0.09; *P*<.001; n=1980; k=8). In addition, iCBT was associated with a higher treatment response (ie, 50% reduction of depressive symptoms) compared with control conditions (b=0.36; 95% CI 0.12-0.60; *P*=.008; n=1980; k=8). Based on model comparisons, these treatment effects were modeled as fixed effects (ie, as homogeneous treatment effects).

iCBT interventions were associated with reduced severity of anxiety compared with controls (b=−0.16; 95% CI −0.26 to −0.06; *P*=.002; n=1451; k=4). In contrast, there was no significant difference in treatment response (ie, 50% reduction of anxiety symptoms) compared with control conditions (b=0.35; 95% CI 0.02-0.67; *P*=.08; n=1451; k=4) after correcting for multiple testing. Based on model comparisons, treatment effects were modeled as fixed effects.

We did not identify a reduction of hopelessness in iCBT compared with control conditions (b=−0.24; 95% CI −0.48 to 0.00; *P*=.11; n=1785; k=5). Similarly, there was no significant effect on treatment response (ie, 50% reduction in hopelessness) in iCBT compared with controls (b=0.56; 95% CI −0.12 to 1.25; *P*=.21; n=1785; k=5). In hopelessness models, treatment effects were modeled as random effects (ie, heterogeneous treatment effects) based on model comparisons.

For a descriptive overview of changes in symptom severity and treatment response, see Table 3. In iCBT, rates of treatment response (ie, 50% symptom reduction) were 21.3% (211/990) regarding depression (controls: 162/990, 16.3%), 16.4% (120/729) regarding anxiety (controls: 88/722, 12.2%), and 19.9% (177/888) regarding hopelessness (controls: 131/897, 14.6%).

**Table 3:** Descriptive symptom changes from baseline to postintervention.^a^

		iCBT^b^ conditions		Control conditions		Total sample	
		Participant, n	Value	Participant, n	Value	Participant, n	Value
**Changes in symptom severity from baseline to postintervention^c^, mean (SD)**							
	Depression (k=8)^d^	990	−0.55 (0.90)	990	−0.39 (0.88)	1980	−0.47 (0.89)
	Anxiety (k=4)	729	−0.46 (0.95)	722	−0.30 (0.92)	1451	−0.38 (0.94)
	Hopelessness (k=5)	888	−0.52 (0.96)	897	−0.29 (0.87)	1785	−0.40 (0.92)
**Treatment response (50% symptom reduction), n (%)**							
	Depression (k=8)	990	211 (21.3)	990	162 (16.3)	1980	373 (18.8)
	Anxiety (k=4)	729	120 (16.4)	722	88 (12.2)	1451	208 (14.3)
	Hopelessness (k=5)	888	177 (19.9)	897	131 (14.6)	1785	308 (17.7)

^a^These analyses are based on the imputed data.

^b^iCBT: internet-based cognitive behavioral therapy.

^c^Scaled to the study-specific variance as different measures were used.

^d^k: the number of studies reporting the respective outcome.

### Conventional Meta-analyses

In the conventional random-effects meta-analyses, we found a reduction of depressive symptoms compared with control conditions (*g*=−0.30; 95% CI −0.43 to −0.17; *P*<.001; *I*^2^=40.6%; n=2074; k=9). The meta-analysis indicated no significant reduction of anxiety compared with controls (*g*=−0.16; 95% CI −0.36 to 0.04; *P*=0.11; *I*^2^=63.9%; n=1513; k=5). Furthermore, the meta-analysis on hopelessness revealed no significant reduction compared with control conditions (*g*=−0.27; 95% CI −0.56 to 0.02; *P*=.06; *I*^2^=85.8%; n=1848; k=6). In all 3 meta-analyses, there were no significant subgroup differences between the studies that provided IPD and the study that did not [39].

### Complete Case Analyses

The sensitivity analyses based on complete observations revealed identical results as the analyses based on imputed data, except for the following: (1) no difference between iCBT and control conditions at postintervention was observed in the complete observation analyses with respect to anxiety severity and (2) the likelihood ratio test for model comparison indicated a homogeneous treatment effect with regard to severity of hopelessness (*P*=.053). The homogenous model indicated a significant reduction in hopelessness severity in iCBT compared with controls (b=−0.25; 95% CI −0.36 to −0.14; *P*<.001; n=1031; k=5), whereas the heterogeneous model did not (b=−0.27; 95% CI −0.57 to 0.00; *P*=.26; n=1031; k=5). A summary of the results of the sensitivity analyses based on complete observations can be found in the table in Multimedia Appendix 3.

### Risk of Bias

The risk of bias tables for the outcomes of depression, anxiety, and hopelessness are displayed in Multimedia Appendix 4. The main source of potential bias in this IPDMA was bias due to missing outcome data across all outcomes. The rates of missing outcome data were >30% (or the differences in missing outcome rates between conditions were >10%) in 4 out of 8 trials with a depression measure, in 3 out of 4 trials with an anxiety measure, and in 3 out of 5 trials with a hopelessness measure. The risk of bias assessment was mostly rated as low in the domains of bias arising from the randomization process, due to deviations from intended interventions, and in the measurement of the outcome. The potential for bias in the selection of the reported results was rated as low for all trials, reflecting the predefined study procedure and analysis strategy in this IPDMA. Additional checks of the IPD revealed that in most trials, there were no range restrictions in the measures of depression, anxiety, and hopelessness, indicating a low risk of bias due to range restrictions.

### Dropout

At postintervention, 771 (38.9%) out of 1980 participants did not provide data on suicidal ideation; here, we selected suicidal ideation as a proxy for dropout because this was the primary outcome of most included trials. In total, 335 (33.8%) out of 990 participants dropped out from control conditions, and 436 (44%) out 990 participants dropped out from the iCBT conditions.

## Discussion

This is the first IPDMA examining the effects of iCBT for individuals with suicidal ideation or behaviors on depression, anxiety, and hopelessness. We identified significant effects on depression, but no consistent effect on anxiety and no effect on hopelessness. It should be noted that participants predominantly received unguided self-help interventions, which is often associated with a reduced effect size compared to digital interventions with human support [41,42].

Regarding personalized treatment planning, the results suggest that while a reduction in depressive symptoms is likely to be achieved by suicidal ideation–specific iCBT, patients with comorbid anxiety and hopelessness may benefit from additional treatment components. Generally, digital means can be effective in treating anxiety symptoms [43]. In behavioral treatment of anxiety, specific treatment elements, including exposure, are considered essential for successful treatment [44]. Anxiety symptoms likely persisted because such best-practice approaches were not present in the included interventions. In contrast, studies on interventions to reduce hopelessness are scarce [45]. However, secondary outcome analyses suggest an effect of face-to-face CBT for depression [46,47] and suicidal ideation [24-26] on hopelessness. Additionally, no clinical study has yet investigated the effects of a digital intervention specifically targeting hopelessness.

However, it is not clear whether treating comorbid symptoms will lead to better treatment outcomes for suicidal ideation. An RCT conducted by Batterham et al [33] did not indicate benefits from tailoring iCBT to individual symptoms. An investigation on the consistency of trajectories of suicidal ideation and depression in an RCT on iCBT for people with suicidal ideation found that changes in depression were related but separate from changes in suicidal thinking [48]. Additionally, transdiagnostic digital interventions have been shown to be equally effective compared to disorder-specific interventions, suggesting that greater specificity of interventions does not necessarily lead to better outcomes [49,50].

The effects found on depression cannot be clearly attributed to specific intervention components. While the intervention modules resemble those used in iCBT for people with depression (eg, cognitive restructuring, psychoeducation on emotions, problem-solving, or mindfulness-based components) [41], the actual content related to specific suicidal symptoms likely differs. For example, a cognitive restructuring intervention for suicidal ideation would have different contents compared to those for depression. Conversely, Torok and colleagues [7] found no effects of digital interventions designed for the treatment of depression on suicidal ideation. However, this conventional meta-analysis was based on aggregated data from only 6 RCTs, which might not have been sufficiently powered to detect an effect on suicidality [7].

In future studies, it would be worthwhile to investigate whether direct treatment of comorbid symptoms of anxiety and hopelessness could lead to improved outcomes for suicidal ideation. This might be particularly relevant for hopelessness, as hopelessness is considered a key driver for suicidal ideation in some suicide theories [51-54]. Furthermore, a lack of hope, which may be associated with reduced treatment expectancy, is associated with reduced effects of psychotherapy [55-59]. Prior meta-analyses investigating face-to-face CBT for suicidal ideation have shown effects on hopelessness [24-26]. However, the quality of evidence in our study and previous meta-analyses does not yet support drawing conclusions about whether the difference in results is attributable to the mode of delivery (ie, face-to-face vs web based). Specific effects of certain intervention components could be examined through component network analysis [60]. However, for such analyses, more studies are needed, and future studies should include detailed information about intervention components as well as specific data on which patients actively worked on which intervention components. It is also possible that anxiety and hopelessness may act as effect-mediating variables for suicidal ideation and behaviors in certain individuals. Hence, future studies should adhere to central recommendations for research on change processes [61] and provide the necessary data to enable mediation analyses in the context of IPDMA [62].

One possible reason for the limited effects of iCBT interventions for suicidal ideation on both suicidal ideation [11] and related mental health problems found in this study may be that existing digital interventions have not adequately considered the complexity of suicidality [63]. As outlined in the *Introduction* section, suicidal ideation is a complex phenomenon that can manifest both independently and in association with various mental and physical health conditions [13-18]. Even within the same diagnosis, certain symptom clusters seem to be more strongly associated with suicidal ideation than others [64]. In addition, complex network theories suggest that various additional factors, including genetic, metabolic, social, and environmental factors, may be responsible for the persistence of suicidality [20,65]. Moreover, the temporal dynamics of suicidal ideation and associated risk factors within days or even hours should be considered [66-68]. Ecological momentary assessment and mobile-sensing studies may be a fruitful next step in this regard to identify interacting variables of individual networks with higher temporal resolution and allow for the examination of between-subject and within-subject differences [69,70]. Comprehensive assessments that take into account various factors contributing to an individual's suicidal ideation network may then inform the development of personalized interventions, potentially leading to more effective and sustainable treatment outcomes [23]. Just-in-time-adaptive interventions may be able to address the temporal dynamics in this context [71].

Several limitations should be considered when interpreting the findings of this IPDMA. First, this is a meta-analysis of secondary outcomes from primary investigations. This renders effect size estimates prone to bias due to the considerable heterogeneity between the individual trials and because not all trials investigated every outcome. This data basis therefore did not allow qualified moderator analyses to be performed. Second, the results may be susceptible to attrition bias as we used multiple imputation (underlying the missing at random assumption) for missing data, which was present to a substantial degree. Third, we could not examine the influence of guidance on intervention effectiveness because only one relevant guided trial with IPD was included. However, the effectiveness in this trial did not differ significantly from the trials on unguided interventions. Fourth, it was not possible to assess the effects of specific intervention components due to the low number of included trials. In contrast, a larger meta-analysis of digital interventions for depression revealed specific advantages or harms of distinct intervention components [60]. Fifth, partially attributable to the smaller number of available studies, it remains unclear whether the effects on hopelessness are heterogeneous or homogeneous, which implies some level of uncertainty in the models and could contribute to the elucidation of why results were not consistent. Sixth, items related to suicidal ideation were included when calculating the total score for depression measures. This might have inflated the intervention effect on depressive symptoms. Seventh, the studies we included have little data from marginalized groups or low-income and middle-income countries, which reduces the validity of our results for more diverse populations. For example, evidence suggests that suicidality is less strongly associated with mental disorders in low-income and middle-income countries compared to high-income countries [72]. In addition to clinical factors, it is pertinent to explore the impact of sociopolitical factors, poverties, and the cultural and social determinants of health when examining suicide in diverse populations [72]. Eighth, using a cutoff (ie, 50% symptom reduction) to investigate treatment response may come with statistical pitfalls and has to be interpreted with caution [73,74]. This includes the proportion of responders as well as the investigations of treatment effects compared to control conditions. Nevertheless, the IPDMA of treatment response usually showed results that were consistent with the continuous outcome and may therefore be viewed as sensitivity analyses.

In conclusion, this IPDMA of RCTs investigating the effectiveness of iCBT for people with suicidal ideation revealed significant effects on depression outcomes, but their effectiveness on anxiety and hopelessness is limited. These findings suggest that to improve care, patients with comorbid anxiety and hopelessness may require additional treatment components. To advance the field of digital interventions for the treatment of people with suicidal ideation, studies that use technology for longitudinal monitoring with higher temporal resolution and consider a broader spectrum of factors influencing the individual suicidal ideation network could lay the groundwork.

## Appendix

Multimedia Appendix 1 PRISMA-IPD (Preferred Reporting Items for Systematic Reviews and Meta-Analyses–Individual Participant Data) checklist.

Multimedia Appendix 2 Electronic search strategy.

Multimedia Appendix 3 Results of complete cases analysis.

Multimedia Appendix 4 Risk of bias ratings' secondary outcomes in meta-analysis of individual participant data.

## Abbreviations

CBT: cognitive behavioral therapy
iCBT: internet-based cognitive behavioral therapy
IPD: individual participant data
IPDMA: meta-analysis of individual participant data
OSF: Open Science Framework
PRISMA: Preferred Reporting Items for Systematic Reviews and Meta-Analyses
RCT: randomized controlled trial

## Data Availability

The analysis R codes are available on the internet at Open Science Framework (https://osf.io/mvnjk) with publication. Individual participant data cannot be provided due to confidentiality agreements.

## References

[1] (2022). World Mental Health Report: transforming mental health for all. World Health Organization.

[2] Franklin JC, Ribeiro JD, Fox KR, Bentley KH, Kleiman EM, Huang X, Musacchio KM, Jaroszewski AC, Chang BP, Nock MK (2017). Risk factors for suicidal thoughts and behaviors: a meta-analysis of 50 years of research. Psychol Bull.

[3] Brown GK, Beck AT, Steer RA, Grisham JR (2000). Risk factors for suicide in psychiatric outpatients: a 20-year prospective study. J Consult Clin Psychol.

[4] Brown GK, Steer RA, Henriques GR, Beck AT (2005). The internal struggle between the wish to die and the wish to live: a risk factor for suicide. Am J Psychiatry.

[5] Bruffaerts R, Demyttenaere K, Hwang I, Chiu W-T, Sampson N, Kessler RC, Alonso J, Borges G, de Girolamo G, de Graaf R, Florescu S, Gureje O, Hu C, Karam EG, Kawakami N, Kostyuchenko S, Kovess-Masfety V, Lee S, Levinson D, Matschinger H, Posada-Villa J, Sagar R, Scott KM, Stein DJ, Tomov T, Viana MC, Nock MK (2011). Treatment of suicidal people around the world. Br J Psychiatry.

[6] Reily NM, Tang S, Batterham PJ, Aadam B, Draper B, Shand F, Han J, Nicholas A, Christensen H (2023). Help-seeking and barriers to service use amongst men with past-year suicidal ideation and not in contact with mental health services. Arch Suicide Res.

[7] Torok M, Han J, Baker S, Werner-Seidler A, Wong I, Larsen ME, Christensen H (2020). Suicide prevention using self-guided digital interventions: a systematic review and meta-analysis of randomised controlled trials. Lancet Digit Health.

[8] Melia R, Francis K, Hickey E, Bogue J, Duggan J, O'Sullivan M, Young K (2020). Mobile health technology interventions for suicide prevention: systematic review. JMIR Mhealth Uhealth.

[9] Stefanopoulou E, Hogarth H, Taylor M, Russell-Haines K, Lewis D, Larkin J (2020). Are digital interventions effective in reducing suicidal ideation and self-harm? A systematic review. J Ment Health.

[10] Witt K, Spittal MJ, Carter G, Pirkis J, Hetrick S, Currier D, Robinson J, Milner A (2017). Effectiveness of online and mobile telephone applications ('apps') for the self-management of suicidal ideation and self-harm: a systematic review and meta-analysis. BMC Psychiatry.

[11] Büscher R, Beisemann M, Doebler P, Micklitz HM, Kerkhof A, Cuijpers P, Batterham PJ, Calear AL, Christensen H, De Jaegere E, Domhardt M, Erlangsen A, van Bergeijk OE, Hill R, Lungu A, Mühlmann C, Pettit JW, Portzky G, Steubl LS, van Spijker BAJ, Tighe J, Werner-Seidler A, Wilks CR, Sander LB (2022). Digital cognitive-behavioural therapy to reduce suicidal ideation and behaviours: a systematic review and meta-analysis of individual participant data. Evid Based Ment Health.

[12] Meerwijk EL, Parekh A, Oquendo MA, Allen IE, Franck LS, Lee KA (2016). Direct versus indirect psychosocial and behavioural interventions to prevent suicide and suicide attempts: a systematic review and meta-analysis. Lancet Psychiatry.

[13] Batterham PJ, Calear AL, Christensen H, Carragher N, Sunderland M (2018). Independent effects of mental disorders on suicidal behavior in the community. Suicide Life Threat Behav.

[14] Sarchiapone M, Carli V, Cuomo C, Roy A (2007). Childhood trauma and suicide attempts in patients with unipolar depression. Depress Anxiety.

[15] Goodwin RD, Kroenke K, Hoven CW, Spitzer RL (2003). Major depression, physical illness, and suicidal ideation in primary care. Psychosom Med.

[16] Scott KM, Hwang I, Chiu W-T, Kessler RC, Sampson NA, Angermeyer M, Beautrais A, Borges G, Bruffaerts R, de Graaf R, Florescu S, Fukao A, Haro JM, Hu C, Kovess V, Levinson D, Posada-Villa J, Scocco P, Nock MK (2010). Chronic physical conditions and their association with first onset of suicidal behavior in the world mental health surveys. Psychosom Med.

[17] Bachmann S (2018). Epidemiology of suicide and the psychiatric perspective. Int J Environ Res Public Health.

[18] Nock MK, Hwang I, Sampson NA, Kessler RC (2010). Mental disorders, comorbidity and suicidal behavior: results from the national comorbidity survey replication. Mol Psychiatry.

[19] Ferrari AJ, Norman RE, Freedman G, Baxter AJ, Pirkis JE, Harris MG, Page A, Carnahan E, Degenhardt L, Vos T, Whiteford HA (2014). The burden attributable to mental and substance use disorders as risk factors for suicide: findings from the Global Burden of Disease Study 2010. PLoS One.

[20] de Beurs D, Bockting C, Kerkhof A, Scheepers F, O'Connor R, Penninx B, van de Leemput I (2021). A network perspective on suicidal behavior: understanding suicidality as a complex system. Suicide Life Threat Behav.

[21] Borsboom D, Cramer AOJ (2013). Network analysis: an integrative approach to the structure of psychopathology. Annu Rev Clin Psychol.

[22] Liu RT, Walsh RFL, Sheehan AE, Cheek SM, Sanzari CM (2022). Prevalence and correlates of suicide and nonsuicidal self-injury in children: a systematic review and meta-analysis. JAMA Psychiatry.

[23] Cohen Z, Delgadillo J, DeRubeis R, Castonguay LG, Barkham M, Lutz W (2021). Personalized treatment approaches. Bergin and Garfield's Handbook of Psychotherapy and Behavior Change, 50th Anniversary Edition.

[24] Witt KG, Hetrick SE, Rajaram G, Hazell P, Taylor Salisbury TL, Townsend E, Hawton K (2021). Psychosocial interventions for self-harm in adults. Cochrane Database Syst Rev.

[25] Tarrier N, Taylor K, Gooding P (2008). Cognitive-behavioral interventions to reduce suicide behavior: a systematic review and meta-analysis. Behav Modif.

[26] D'Anci KE, Uhl S, Giradi G, Martin C (2019). Treatments for the prevention and management of suicide: a systematic review. Ann Intern Med.

[27] Clarke MJ (2005). Individual patient data meta-analyses. Best Pract Res Clin Obstet Gynaecol.

[28] Smith CT, Marcucci M, Nolan SJ, Iorio A, Sudell M, Riley R, Rovers MM, Williamson PR (2016). Individual participant data meta-analyses compared with meta-analyses based on aggregate data. Cochrane Database Syst Rev.

[29] Debray TPA, Moons KGM, Abo-Zaid GMA, Koffijberg H, Riley RD (2013). Individual participant data meta-analysis for a binary outcome: one-stage or two-stage?. PLoS One.

[30] Frank E, Prien RF, Jarrett RB, Keller MB, Kupfer DJ, Lavori PW, Rush AJ, Weissman MM (1991). Conceptualization and rationale for consensus definitions of terms in major depressive disorder. Remission, recovery, relapse, and recurrence. Arch Gen Psychiatry.

[31] van Spijker BAJ, van Straten SA, Kerkhof AJFM (2014). Effectiveness of online self-help for suicidal thoughts: results of a randomised controlled trial. PLoS One.

[32] Tighe J, Shand F, Ridani R, Mackinnon A, De La Mata N, Christensen H (2017). Ibobbly mobile health intervention for suicide prevention in Australian Indigenous youth: a pilot randomised controlled trial. BMJ Open.

[33] Batterham PJ, Calear AL, Farrer L, McCallum SM, Cheng VWS (2018). FitMindKit: randomised controlled trial of an automatically tailored online program for mood, anxiety, substance use and suicidality. Internet Interv.

[34] Mühlmann C, Madsen T, Hjorthøj C, Forman JL, Kerkhof AJFM, Nordentoft M, Erlangsen A (2021). Effectiveness of an internet-based self-help therapy program for suicidal ideation with follow-up at 6 months: results of a randomized controlled trial. J Clin Psychiatry.

[35] Eylem O, van Straten A, de Wit L, Rathod S, Bhui K, Kerkhof AJFM (2021). Reducing suicidal ideation among Turkish migrants in the Netherlands and in the UK: the feasibility of a randomised controlled trial of a guided online intervention. Pilot Feasibility Stud.

[36] Hill RM, Pettit JW (2019). Pilot randomized controlled trial of LEAP: a selective preventive intervention to reduce adolescents' perceived burdensomeness. J Clin Child Adolesc Psychol.

[37] De Jaegere E, van Landschoot R, van Heeringen K, van Spijker BAJ, Kerkhof AJFM, Mokkenstorm JK, Portzky G (2019). The online treatment of suicidal ideation: a randomised controlled trial of an unguided web-based intervention. Behav Res Ther.

[38] van Spijker BA, Werner-Seidler A, Batterham PJ, Mackinnon A, Calear AL, Gosling JA, Reynolds J, Kerkhof AJ, Solomon D, Shand F, Christensen H (2018). Effectiveness of a web-based self-help program for suicidal thinking in an Australian community sample: randomized controlled trial. J Med Internet Res.

[39] Hetrick SE, Yuen HP, Bailey E, Cox GR, Templer K, Rice SM, Bendall S, Robinson J (2017). Internet-based cognitive behavioural therapy for young people with suicide-related behaviour (Reframe-IT): a randomised controlled trial. Evid Based Ment Health.

[40] van Spijker BAJ, van Straten A, Kerkhof AJFM (2010). The effectiveness of a web-based self-help intervention to reduce suicidal thoughts: a randomized controlled trial. Trials.

[41] Moshe I, Terhorst Y, Philippi P, Domhardt M, Cuijpers P, Cristea I, Pulkki-Råback L, Baumeister H, Sander LB (2021). Digital interventions for the treatment of depression: a meta-analytic review. Psychol Bull.

[42] Karyotaki E, Efthimiou O, Miguel C, Bermpohl FMG, Furukawa TA, Cuijpers P, Individual Patient Data Meta-Analyses for Depression (IPDMA-DE) Collaboration (2021). Internet-based cognitive behavioral therapy for depression: a systematic review and individual patient data network meta-analysis. JAMA Psychiatry.

[43] Pauley D, Cuijpers P, Papola D, Miguel C, Karyotaki E (2023). Two decades of digital interventions for anxiety disorders: a systematic review and meta-analysis of treatment effectiveness. Psychol Med.

[44] Kaczkurkin AN, Foa EB (2015). Cognitive-behavioral therapy for anxiety disorders: an update on the empirical evidence. Dialogues Clin Neurosci.

[45] Hernandez SC, Overholser JC (2021). A systematic review of interventions for hope/hopelessness in older adults. Clin Gerontol.

[46] Cuijpers P, de Beurs DP, van Spijker BAJ, Berking M, Andersson G, Kerkhof AJFM (2013). The effects of psychotherapy for adult depression on suicidality and hopelessness: a systematic review and meta-analysis. J Affect Disord.

[47] Handley TE, Kay-Lambkin FJ, Baker AL, Lewin TJ, Kelly BJ, Inder KJ, Attia JR, Kavanagh DJ (2013). Incidental treatment effects of CBT on suicidal ideation and hopelessness. J Affect Disord.

[48] Batterham PJ, van Spijker BAJ, Mackinnon AJ, Calear AL, Wong Q, Christensen H (2019). Consistency of trajectories of suicidal ideation and depression symptoms: evidence from a randomized controlled trial. Depress Anxiety.

[49] Fogliati VJ, Dear BF, Staples LG, Terides MD, Sheehan J, Johnston L, Kayrouz R, Dear R, McEvoy PM, Titov N (2016). Disorder-specific versus transdiagnostic and clinician-guided versus self-guided internet-delivered treatment for panic disorder and comorbid disorders: a randomized controlled trial. J Anxiety Disord.

[50] Dear BF, Staples LG, Terides MD, Fogliati VJ, Sheehan J, Johnston L, Kayrouz R, Dear R, McEvoy PM, Titov N (2016). Transdiagnostic versus disorder-specific and clinician-guided versus self-guided internet-delivered treatment for social anxiety disorder and comorbid disorders: a randomized controlled trial. J Anxiety Disord.

[51] Joiner TE (2005). Why People Die by Suicide.

[52] Klonsky ED, May AM (2015). The three-step theory (3ST): a new theory of suicide rooted in the “ideation-to-action” framework. Int J Cogn Ther.

[53] Kuo W-H, Gallo JJ, Eaton WW (2004). Hopelessness, depression, substance disorder, and suicidality—a 13-year community-based study. Soc Psychiatry Psychiatr Epidemiol.

[54] Beck AT, Steer RA, Beck JS, Newman CF (1993). Hopelessness, depression, suicidal ideation, and clinical diagnosis of depression. Suicide Life Threat Behav.

[55] Greenberg RP, Constantino MJ, Bruce N (2006). Are patient expectations still relevant for psychotherapy process and outcome?. Clin Psychol Rev.

[56] Meyer B, Pilkonis PA, Krupnick JL, Egan MK, Simmens SJ, Sotsky SM (2002). Treatment expectancies, patient alliance, and outcome: further analyses from the national institute of mental health treatment of depression collaborative research program. J Consult Clin Psychol.

[57] Irving LM, Snyder CR, Cheavens J, Gravel L, Hanke J, Hilberg P, Nelson N (2004). The relationships between hope and outcomes at the pretreatment, beginning, and later phases of psychotherapy. J. Psychother. Integr.

[58] Constantino MJ, Arnkoff DB, Glass CR, Ametrano RM, Smith JZ (2011). Expectations. J Clin Psychol.

[59] Larsen DJ, Stege R (2010). Hope-focused practices during early psychotherapy sessions: part I: implicit approaches. J Psychother Integr.

[60] Furukawa TA, Suganuma A, Ostinelli EG, Andersson G, Beevers CG, Shumake J, Berger T, Boele FW, Buntrock C, Carlbring P, Choi I, Christensen H, Mackinnon A, Dahne J, Huibers MJH, Ebert DD, Farrer L, Forand NR, Strunk DR, Ezawa ID, Forsell E, Kaldo V, Geraedts A, Gilbody S, Littlewood E, Brabyn S, Hadjistavropoulos HD, Schneider LH, Johansson R, Kenter R, Kivi M, Björkelund C, Kleiboer A, Riper H, Klein JP, Schröder J, Meyer B, Moritz S, Bücker L, Lintvedt O, Johansson P, Lundgren J, Milgrom J, Gemmill AW, Mohr DC, Montero-Marin J, Garcia-Campayo J, Nobis S, Zarski A-C, O'Moore K, Williams AD, Newby JM, Perini S, Phillips R, Schneider J, Pots W, Pugh NE, Richards D, Rosso IM, Rauch SL, Sheeber LB, Smith J, Spek V, Pop VJ, Ünlü B, van Bastelaar KMP, van Luenen S, Garnefski N, Kraaij V, Vernmark K, Warmerdam L, van Straten A, Zagorscak P, Knaevelsrud C, Heinrich M, Miguel C, Cipriani A, Efthimiou O, Karyotaki E, Cuijpers P (2021). Dismantling, optimising, and personalising internet cognitive behavioural therapy for depression: a systematic review and component network meta-analysis using individual participant data. Lancet Psychiat.

[61] Domhardt M, Steubl L, Boettcher J, Buntrock C, Karyotaki E, Ebert DD, Cuijpers P, Baumeister H (2021). Mediators and mechanisms of change in internet- and mobile-based interventions for depression: a systematic review. Clin Psychol Rev.

[62] Domhardt M, Grund S, Mayer A, Büscher R, Ebert DD, Sander LB, Karyotaki E, Cuijpers P, Baumeister H (2022). Unveiling mechanisms of change in digital interventions for depression: study protocol for a systematic review and individual participant data meta-analysis. Front Psychiatry.

[63] Millner AJ, Robinaugh DJ, Nock MK (2020). Advancing the understanding of suicide: the need for formal theory and rigorous descriptive research. Trends Cogn Sci.

[64] Keilp JG, Ellis SP, Gorlyn M, Burke AK, Oquendo MA, Mann JJ, Grunebaum MF (2018). Suicidal ideation declines with improvement in the subjective symptoms of major depression. J Affect Disord.

[65] van Heeringen K, Mann JJ (2014). The neurobiology of suicide. Lancet Psychiatry.

[66] Bryan CJ, Rozek DC, Butner J, Rudd MD (2019). Patterns of change in suicide ideation signal the recurrence of suicide attempts among high-risk psychiatric outpatients. Behav Res Ther.

[67] Hallensleben N, Spangenberg L, Forkmann T, Rath D, Hegerl U, Kersting A, Kallert TW, Glaesmer H (2018). Investigating the dynamics of suicidal ideation. Crisis.

[68] Kleiman EM, Nock MK (2018). Real-time assessment of suicidal thoughts and behaviors. Curr Opin Psychol.

[69] Forkmann T, Spangenberg L, Rath D, Hallensleben N, Hegerl U, Kersting A, Glaesmer H (2018). Assessing suicidality in real time: a psychometric evaluation of self-report items for the assessment of suicidal ideation and its proximal risk factors using ecological momentary assessments. J Abnorm Psychol.

[70] Bolger N, Laurenceau JP (2013). Intensive longitudinal methods: an introduction to diary and experience sampling research.

[71] Torous J, Larsen ME, Depp C, Cosco TD, Barnett I, Nock MK, Firth J (2018). Smartphones, sensors, and machine learning to advance real-time prediction and interventions for suicide prevention: a review of current progress and next steps. Curr Psychiatry Rep.

[72] Knipe D, Padmanathan P, Newton-Howes G, Chan LF, Kapur N (2022). Suicide and self-harm. Lancet.

[73] Senn S (2009). Three things that every medical writer should know about statistics. The Write Stuff.

[74] Senn S (2016). Mastering variation: variance components and personalised medicine. Stat Med.

