# Supplementary material for: The Effects of Internet-Based Cognitive Behavioral Therapy for Suicidal Ideation or Behaviors on Depression, Anxiety, and Hopelessness in Individuals With Suicidal Ideation: Systematic Review and Meta-Analysis of Individual Participant Data
Source: J Med Internet Res. 2023 Jun 26;25:e46771. doi: 10.2196/46771 (PMC10337381; doi:10.2196/46771)
Supplement: Multimedia Appendix 2 [file jmir_v25i1e46771_app2.docx]

**Electronic Search Strategy**

Note: The full search strategy including search strings for CENTRAL, PsyINFO and Embase was published in the study protocol (doi:10.3390/ijerph17145179).

Search string for Pubmed:
(computers[MeSH Terms] OR software[MeSH Terms] OR internet[MeSH Terms] OR web browser[MeSH Terms] OR technology[MeSH Terms] OR cell phone[MeSH Terms] OR mobile applications[MeSH Terms] OR therapy, computer-assisted [MeSH Terms] OR telemedicine[MeSH Terms] OR telerehabiliation[MeSH Terms] OR medical informatics[MeSH Terms] OR distance counseling[MeSH Terms] OR technolog*[Title/Abstract] OR software[Title/Abstract] OR web[Title/Abstract] OR “app-based”[Title/Abstract] OR “app based”[Title/Abstract] OR internet[Title/Abstract] OR online[Title/Abstract] OR computer*[Title/Abstract] OR
cyber[Title/Abstract] OR electronic[Title/Abstract] OR “world wide web”[Title/Abstract] OR
www[Title/Abstract] OR net[Title/Abstract] OR digital[Title/Abstract] OR virtual[Title/Abstract] OR
website[Title/Abstract] OR chat[Title/Abstract] OR forum[Title/Abstract] OR e-mail[Title/Abstract] OR
email[Title/Abstract] OR SMS[Title/Abstract] OR “text messag*”[Title/Abstract] OR
textmessag*[Title/Abstract] OR mobile[Title/Abstract] OR smartphone[Title/Abstract] OR
phone[Title/Abstract] OR e-therap*[Title/Abstract] OR “e-mental health”[Title/Abstract] OR “emental health”[Title/Abstract] OR e-health[Title/Abstract] OR ehealth[Title/Abstract] OR mhealth[Title/Abstract] OR m-health[Title/Abstract] OR tele-care[Title/Abstract] OR telecare[Title/Abstract] OR tele-health[Title/Abstract] OR telehealth[Title/Abstract] OR tele-medicine[Title/Abstract] OR telemedicine[Title/Abstract] OR tele-rehabilitation[Title/Abstract] OR telerehabilitation[Title/Abstract] OR telephone [Title/Abstract] OR iCBT[Title/Abstract] OR i-CBT[Title/Abstract] OR cCBT[Title/Abstract] OR c-CBT[Title/Abstract] OR "personal digital assist*"[Title/Abstract] OR PDA[Title/Abstract] OR "cell* phone*"[Title/Abstract]) AND (suicide[MeSH Terms] OR “self-injurious behavior”[MeSH Terms] OR “suicidal ideation”[ MeSH Terms] OR “suicide, attempted”[MeSH Terms] OR suicid*[ Title/Abstract] OR self-injur*[Title/Abstract] OR selfinjur*[Title/Abstract] OR self-harm[Title/Abstract] OR selfharm[Title/Abstract] OR self- mutilation[Title/Abstract] OR selfmutilation[Title/Abstract] OR auto-mutilation[Title/Abstract] OR automutilation[Title/Abstract]) AND (“randomized controlled trials as topic”[MeSH Terms] OR “clinical trials as topic”[MeSH Terms] OR “randomized controlled trial”[Publication Type] OR “controlled clinical trial”[Publication Type] OR “clinical trial”[Publication Type] OR “clinical trial protocol”[Publication Type] OR “clinical study”[Publication Type] OR RCT[Title/Abstract] OR random*[Title/Abstract] OR trial [Title/Abstract])
