# Supplementary material for: The Effects of Internet-Based Cognitive Behavioral Therapy for Suicidal Ideation or Behaviors on Depression, Anxiety, and Hopelessness in Individuals With Suicidal Ideation: Systematic Review and Meta-Analysis of Individual Participant Data
Source: J Med Internet Res. 2023 Jun 26;25:e46771. doi: 10.2196/46771 (PMC10337381; doi:10.2196/46771)
Supplement: Multimedia Appendix 3 [file jmir_v25i1e46771_app3.docx]

**Results Complete Case Analysis**

|  |  | Symptom severity (continuous) | | | Treatment response (50% symptom reduction) | | |
| --- | --- | --- | --- | --- | --- | --- | --- |
|  | n (k)^b^ | b (SE)^c^ | 95% CI^d^ | p | b (SE)^c^ | 95% CI^d^ | p |
| *Effects at post-intervention* |  |  |  |  |  |  |  |
| Depression | 1184 (8) | -0.215 (0.051) | -0.315; -0.116 | <0.001 | 0.412 (0.151) | 0.116; 0.709 | 0.013 |
| Anxiety | 699 (4) | -0.149 (0.072) | -0.288; -0.006 | 0.077 | 0.247 (0.227) | -0.201; 0.696 | 0.554 |
| Hopelessness^e^ | 1031 (5) | -0.247 (0.057)^d^ | -0.359; -0.136^d^ | <0.001^d^ | 0.700 (0.393) | -0.135; 2.096 | 0.149 |

*Note:* These analyses are based on complete observations. p-values have been corrected for multiple testing across the two indices using the Bonferroni correction term. The confidence intervals have not been corrected. ^b^n (k): total number of participants included in the respective analyses (number of studies). ^c^b (SE): b coefficient (standard error). ^d^95% CI: 95% confidence interval. ^e^The treatment effect in the model on severity of hopelessness was modeled as a random effect as indicated in model comparisons; in all other models, the treatment effect was modeled as fixed effects. ^d^The model comparison test for severity of hopelessness was close to significant (p=0.052). The results for the heterogenous model were: b=-0.265; 95%-CI -0.570 to 0.002; p=0.260; n=1031; k=5
