# Supplementary material for: The Effects of Internet-Based Cognitive Behavioral Therapy for Suicidal Ideation or Behaviors on Depression, Anxiety, and Hopelessness in Individuals With Suicidal Ideation: Systematic Review and Meta-Analysis of Individual Participant Data
Source: J Med Internet Res. 2023 Jun 26;25:e46771. doi: 10.2196/46771 (PMC10337381; doi:10.2196/46771)
Supplement: Multimedia Appendix 4 [file jmir_v25i1e46771_app4.docx]

**Risk of Bias Ratings Secondary Outcomes IPD-MA**

In the ratings, we took information from individual participant data into account where relevant.

The following domains were evaluated for each outcome separately: 3 Bias due to missing outcome data (checked missings in IPD per outcome), 4 Bias in measurement of the outcome (checked measure and reliability), 5 Bias in selection of the reported result (checking which measures have been pre-registered).

**Risk of bias depression:**

| **Study** | **1 Bias arising from the randomization process** | **2 Bias due to deviations from intended interventions** | **3 Bias due to missing outcome data** | **4 Bias in measure-ment of the outcome** | **5 Bias in selection of the reported result** |
| --- | --- | --- | --- | --- | --- |
| Batterham 2018 | Low | Low | **High** | Low | Low |
| De Jaegere 2019 | Low | Low | **High** | Low | Low |
| Hill 2016 | Low | Low | Low | Low | Low |
| Mühlmann 2021 | Low | Low | Low | Low | Low |
| Van Spijker 2014 | Low | Low | Low | Low | Low |
| Van Spijker 2018 | **Some concerns** | Low | **High** | Low | Low |
| Tighe 2017 | Low | **High** | Low | **High** | Low |
| Eylem 2021 | Low | Low | **High** | Low | Low |

**Risk of bias anxiety:**

| **Study** | **1 Bias arising from the randomization process** | **2 Bias due to deviations from intended interventions** | **3 Bias due to missing outcome data** | **4 Bias in measure-ment of the outcome** | **5 Bias in selection of the reported result** |
| --- | --- | --- | --- | --- | --- |
| Batterham 2018 | Low | Low | **High** | Low | Low |
| De Jaegere 2019 | Low | Low | **High** | Low | Low |
| Van Spijker 2014 | Low | Low | Low | Low | Low |
| Van Spijker 2018 | **Some concerns** | Low | **High** | Low | Low |

**Risk of bias hopelessness:**

| **Study** | **1 Bias arising from the randomization process** | **2 Bias due to deviations from intended interventions** | **3 Bias due to missing outcome data** | **4 Bias in measure-ment of the outcome** | **5 Bias in selection of the reported result** |
| --- | --- | --- | --- | --- | --- |
| De Jaegere 2019 | Low | Low | **High** | Low | Low |
| Mühlmann 2021 | Low | Low | Low | Low | Low |
| Van Spijker 2014 | Low | Low | Low | Low | Low |
| Van Spijker 2018 | **Some concerns** | Low | **High** | Low | Low |
| Eylem 2021 | Low | Low | **High** | Low | Low |
